# Friction and Wear Performances and Mechanisms of Graphite/Copper Composites Under Electrical Contact in Marine Environments

**DOI:** 10.3390/ma18071516

**Published:** 2025-03-28

**Authors:** Nenghui Wang, Chuanfeng Wang, Wenhu Xu, Weiping Cheng, Haihong Wu, Hongsheng Li

**Affiliations:** 1 School of Instrument Science and Engineering, Southeast University, Nanjing 210096, China; 2 CSSC Jiujiang Marine Equipment (Group) Co., Ltd., Jiujiang 332005, China; 3 School of Advanced Manufacturing, Nanchang University, Nanchang 330031, China

**Keywords:** marine engineering, electrical contacts, graphite/copper composite, current-carrying friction and wear, mating materials

## Abstract

Marine environment-induced apparatus failures have led to substantial losses in marine engineering. Graphite/copper composites, known for their excellent electrical conductivity and wear resistance, are extensively utilized in various electric contact devices. However, research on the current-carrying friction and wear behavior of graphite/copper composites in marine environments is still limited. This study investigates the effects of mating materials, graphite content (30 wt.% and 45 wt.%), and electric voltage on the friction and wear mechanisms of graphite/copper composites in seawater. The results show that under seawater coupled with electricity, no mass loss was observed in the 30 wt.% graphite composites after friction tests against different counterparts. Electric voltage (3 V) affects the composite’s damage mechanism, inducing delamination wear, arc erosion and accelerating corrosion. Specifically, the electricity factor promotes oxidation recreations while inhibiting chlorine formation. Notably, when the composite is paired with gold-coated copper, it undergoes electrochemical reactions, leading to the formation of needle-like copper oxide. These oxides alter the surface morphology, elevate the mass of worn composites, and raise the friction coefficient of the tribopair to approximately 0.3, an increase from 0.2.

## 1. Introduction

Abundant green energy resources in the blue ocean, including tidal, wind, biomass and wave energy, are vital for the sustainable development of human society [1,2,3]. Nevertheless, the unique marine environment significantly affects the functionality of diverse marine engineering equipment. High salinity and humidity can cause the formation of a liquid electrolyte film on the surfaces of electrical contacts in marine environment [4,5]. This film participates in the interactions at the electrical contact interface, resulting in wear and corrosion, which affects the service life of materials and equipment [6,7,8,9].

Graphite/copper composites integrate the lubricity, low thermal expansion and low density of graphite with the high electrical conductivity, thermal conductivity and ductility of copper, thereby exhibiting excellent wear resistance, arc erosion resistance and anti-fusion welding properties [10]. Lin et al. [11] incorporated copper-plated graphite powder into the copper matrix, improving the C–Cu interface bonding, which enables the composites to retain the physical and mechanical properties of the Cu matrix while achieving a lower friction coefficient and wear rate. Song et al. [12] developed copper-graphite gradient composite materials to address the need for optimizing damage in current-carrying friction materials. Andrej et al. [13] investigated the coefficient of thermal expansion of graphite/copper composites and discovered that the coefficient decreases as the graphite volume fraction increases. Kumar et al. [14] found that graphite addition not only decreased the friction coefficient of copper but also improved material strength. Li et al. [15] ascribed the good tribological performance of graphite/copper composites to the synergistic effect of an oxidized friction layer and a graphite lubricating film. Liu et al. [16] investigated the relationship between graphite content and the evolution of contact resistances, revealing that the graphite/copper composites with high graphite content exhibit good resistance to fretting wear but compromise current transmission efficiency. Yang et al. [17,18] studied the effects of graphite content and raw material powder on the current-carrying friction and wear properties of the composite material. Currently, graphite/copper composites are widely used in various mechanical and electrical components. To enhance the tribological properties of copper-graphite composites in applications such as slip-ring assemblies and locomotive pantograph strips, Grandin et al. [19] examined the friction, wear and contact resistance of the graphite/copper composite when rubbed against copper. Xu et al. [20] combined large-size cluster graphite and copper matrix to prepare pantograph sliders with high impact toughness. Wang et al. [21] prepared a Cu-graphite brush using the powder metallurgy process and identified the dominant wear mechanism of the brush under the current as electrical wear.

However, most studies on graphite/copper composites have concentrated on the preparation and friction performance of the material in dry or wet conditions, with limited investigation into their friction behavior in marine environment and the impact of graphite content and current-carrying factors on friction characteristics. Consequently, a thorough study of the friction and wear of copper/graphite composites in the marine environment is of importance for unlocking the application potential of these materials and extending the service life of electrical contact components.

In this study, two graphite/copper composites with varying graphite contents were prepared using the powder metallurgy method. Subsequently, friction pairs of these composites were paired with three mating materials of varying hardness. Current-carrying friction tests were then performed in a simulated seawater environment. Scanning electron microscopy (SEM) and energy dispersive spectroscopy (EDS) were utilized to analyze the microscopic morphology of the wear samples and explore the wear mechanism. The aim of this study is to provide theoretical insights and experimental data to support the selection and application of these materials in marine engineering.

## 2. Materials and Methods

### 2.1. Materials

The addition of a small amount of lead to graphite/copper materials significantly enhances their hardness and improves their friction behavior [22]. Therefore, in this study, some lead was added to the graphite/copper. Copper powder (~400 mesh) was purchased from Beijing Beike New Material Technology Co., Ltd. (Beijing, China), graphite powder (~200 mesh) was provided by Tianjin Gaokexin Material Co., Ltd. (Tianjin, China), and lead powder (~400 mesh) was sourced from Hebei Tebo Metal Material Co., Ltd. (Xingtai, China), all with purities of no less than 99%.

Graphite/copper composites were prepared by the powder metallurgy pressure sintering method. The specific process involved the following steps: First, the raw material powders were weighed. Next, the powders were mixed in a ball mill (MITR YXQM-4L, Changsha, China) with a ball-to-powder ratio of 10:1, using 2 mm copper balls, at a rotational speed of 200 rpm for 24 h. Following mixing, the powders were compressed into rectangular compacts, with a dimension of 4 mm × 4 mm × 25 mm, using a hydraulic press (Riyi FBSY-C05, Wenzhou, China) at a pressure of 300 MPa for 15 s. Finally, the compacts were sintered in a hydrogen furnace (Kejing KSL-1200X, Hefei, China) at 900 °C for 1 h, followed by furnace cooling. Prior studies have shown that incorporating approximately 30~40 wt.% of graphite can improve the current-carrying performance and mechanical strength of graphite/copper composites [16,23]. The chemical element contents of the two graphite/copper composites are detailed in Table 1 below.

The counterpart materials were red copper (T2), zirconium bronze (QCr0.8), and gold-plated red copper (Au/T2 with a gold plating layer thickness of 5 μm). Their hardness values were 70, 150, and 200 HV, respectively.

### 2.2. Methods

The composite hardness was determined by a nanoindenter (Nanotest Vantage, Wrexham, UK). Following fixation and leveling of the sample on the test bench, a load of 200 mN is applied. To enhance the precision of the test results, each sample underwent three repetitions, with all indentations made within the sample matrix.

**Figure 1:**
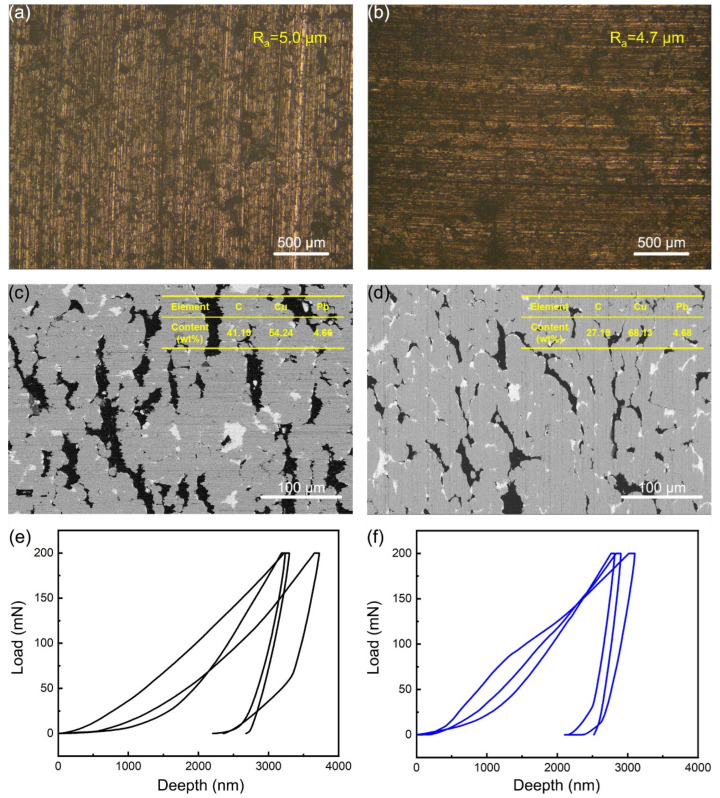
The initial surface topography of sample C1 (**a**) and sample C2 (**b**); The BSE image of sample C1 (**c**) and sample C2 (**d**); The load-depth curves of sample C1 (**e**) and sample C2 (**f**) under loads of 200 mN.

Friction and wear tests were performed using a multifunctional friction and wear testing machine (Tribolab, Billerica, MA, USA) to investigate the wear behavior of graphite/copper in 3.5% NaCl solution under the condition of current-carrying. The dimensions of the graphite/copper composite pin samples were 4 mm × 4 mm × 25 mm, while the counterpart disks had dimensions of Φ70 mm × 5 mm. Prior to the tests, the samples were ground using sandpaper until they achieved similar surface roughness (Figure 1a,b). The tribopairs were immersed in the NaCl solution, and a constant voltage (as depicted in Table 2) was applied during the tests. A load of 5 N and a rotational speed of 75 rpm were applied. The sliding distance was 2986 m, and the sliding time was 1 h. Each set of friction experiments was repeated three times.

The wear rate *W* was calculated using the following formula:

$$W=\frac{m}{s}$$

where *W* is the wear rate (mg/mm^2^), *m* is the wear mass (mg), and *s* is the wear area (mm^2^).

SEM, EDS (Thermo Scientific/Apero 2S HiVac, Waltham, MA, USA), and laser confocal microscope (LSK, Bruker, Billerica, MA, USA) were applied to test the surface morphology, microstructure, and elemental distribution samples. At an accelerating voltage of 15 KV, the step sizes of SEM and EDS were 0.45 μm and 0.31 μm, respectively.

## 3. Results and Discussion

### 3.1. Microstructure and Hardness

The distribution of graphite and Pb components within the copper matrix before testing is shown in Figure 1c,d. These images reveal a gray copper matrix, with the graphite phase appearing as black, elongated islands of varying sizes and the Pb phase as white, worm-like structures uniformly distributed. Additionally, the content of the various elements approximated the preset values. Figure 1e,f show the nanoindentation test results. A greater maximum indentation depth under the same load corresponds to lower material hardness. Due to the softness of graphite, its addition to copper reduces the overall hardness [15,24]. Sample A2 exhibited a lower maximum indentation depth compared to sample A1, indicating that it possesses greater hardness. The hardness of sample A1 is 0.81 GPa, and the hardness of sample A2 is 1.05 GPa.

### 3.2. Friction Coefficient

**Figure 2:**
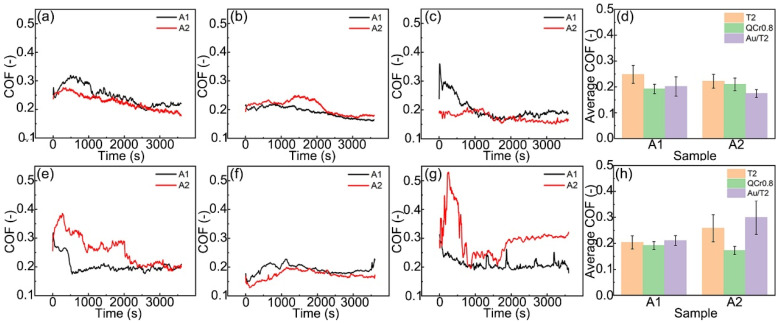
Instantaneous and average friction coefficients of graphite/copper-T2, graphite/copper-QCr0.8, and graphite/copper-Au/T2 (**c**) under C1 (**a**–**d**) and C2 (**e**–**h**) conditions.

Figure 2 illustrates the friction coefficients (COF) of these tribopairs of graphite/copper composites and their counterpart materials under C1 and C2 conditions. As shown in Figure 2a–c, the two graphite/copper samples display similar trends in friction coefficient variations in seawater. As sliding time increases, the friction coefficient decreased and stabilized after the initial run-in period, during which the contact interface achieved an optimal state through plastic deformation and wear. The reduction in friction coefficient over time can be ascribed to the formation of a graphite layer and surface oxidation. As sliding time increases, more graphite is stripped from the copper matrix, leading to the gradual formation of a lubricant film. It reduces the likelihood of direct contact between the tribopairs, causing friction to occur between the lubricating film and the opposing surface. This diminishes the adhesion between metals, leading to a reduction in the friction coefficient [15,16]. Furthermore, during friction, the surface of the composites generally forms many oxides, which facilitates the sliding. This mechanism is in alignment with other established findings [15,25]. Due to the combined effects of graphite and oxides, the friction coefficient deceases as sliding time progresses. Figure 2d demonstrates that the average friction coefficient of these tribopairs decreases as the hardness of the counterpart material increases in a seawater environment.

Furthermore, when a constant voltage was applied to the A1’s tribopair, the time required for the contact interface to stabilize was reduced, and the friction coefficient stabilized at approximately 0.2, consistent with the friction coefficient observed in seawater. Under the effect of voltage, the A2 sample exhibited significant fluctuation in the friction coefficient when paired with Au/T2, and its average friction coefficient increases significantly (Figure 2g,h). Additionally, the friction coefficient of the A2–Au/T2 finally stabilized at approximately 0.3, higher than that of other tribopairs under C1/C2 conditions, indicating a change in the lubricating film at the contact interface (Figure 2h).

### 3.3. Macroscopic Wear Morphology and Wear Rate

**Figure 3:**
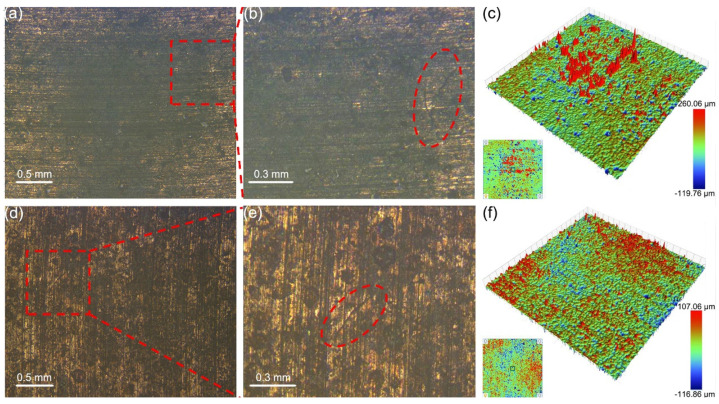
Surface morphology of samples A1 (**a**–**c**) and A2 (**d**–**f**) after friction with T2 under C1 conditions. (**b**) and (**e**) are, respectively, the enlarged image of the area marked with red rectangles in (**a**) and (**d**), with cracks marked by red elliptical dashed lines.

Apparently flat surfaces exhibit some roughness due to micro-asperities. Figure 3 exhibits the wear morphology of the graphite/copper composites after friction tests in seawater. Sample A2 exhibited greater hardness, indicating enhanced deformation resistance [26]. The worn surface of sample A1 was less flat than that of sample A2. These micro-convex bodies on the T2 surface repeatedly pressed and slid against the A1 and A2 samples, causing scratches on their surfaces. During this process, cracks also appeared on the friction surface, and they arise to excessive plastic deformation of the surface. As shown in Figure 3b,e, the cracks on these composites are approximately 0.3–0.4 mm in length. Additionally, wear debris accumulated on the sample surfaces, forming red protrusions in the 3D topography images of A1 (Figure 3c). The maximum height of these protrusions was approximately 260.06 μm. Meanwhile, numerous small pits were observed, resulting from the rolling of abrasive particles during three-body wear [27]. These protrusions and pits increased the roughness of the A1 surface, rendering it more uneven compared to A2.

**Figure 4:**
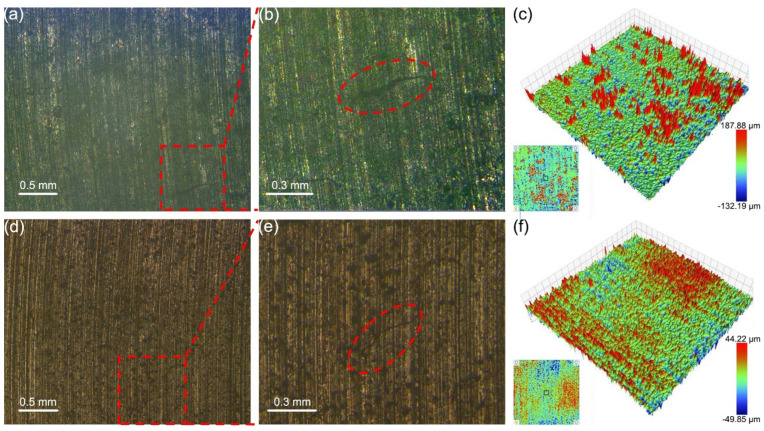
Surface morphology of samples A1 (**a**–**c**) and A2 (**d**–**f**) after friction with QCr0.8 under C1 conditions. (**b**) and (**e**) are, respectively, the enlarged image of the area marked with red rectangles in (**a**) and (**d**), with cracks marked by red elliptical dashed lines.

Figure 4 compares the macroscopic wear morphology of A1 and A2 samples after friction with the harder QCr0.8. For the sample A1, the increased presence of protrusions led to higher surface roughness. Because A2 has higher hardness, during the friction process, A2 exhibited greater resistance to deformation against the surface convex of QCr0.8, resulting in wear morphology and surface roughness similar to those observed after friction with T2.

**Figure 5:**
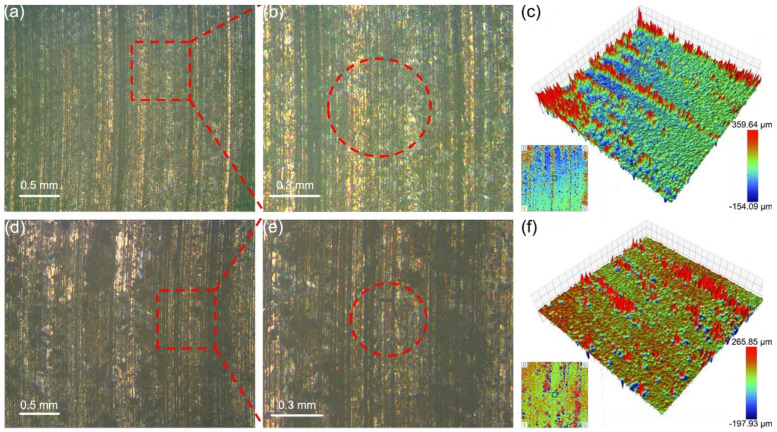
Surface morphology of samples A1 (**a**–**c**) and A2 (**d**–**f**) after friction with Au/T2 under C1 conditions. (**b**) and (**e**) are, respectively, the enlarged image of the area marked with red rectangles in (**a**) and (**d**), with cracks marked by red elliptical dashed lines.

As the hardness of the mating material increases, the wear morphology of the two graphite/copper composites in seawater underwent further changes. Figure 5 demonstrates that the surfaces of A1 and A2 show an increase in protrusions, indicating higher surface roughness after abrasion with the hardest Au/T2. Analysis of the density and height of these protrusions reveals that the surface roughness of A1 is significantly greater than that of A2. Additionally, these small pits observed indicate that there is three-body wear during the friction (Figure 5f).

**Figure 6:**
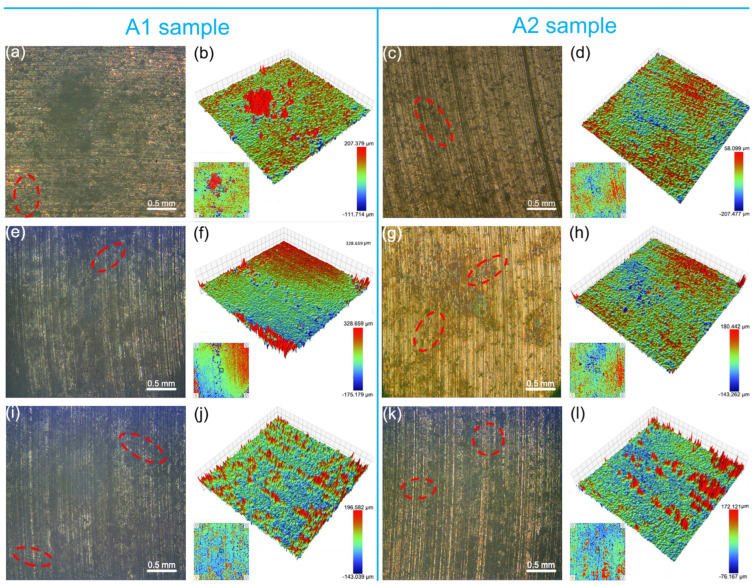
Surface morphology of A1 and A2 samples after friction with T2 (**a**–**d**), QCr0.8 (**e**–**h**), and Au/T2 (**i**–**l**) under C2 conditions, with cracks marked by red elliptical dashed lines.

Figure 6 exhibits the macroscopic wear morphologies of A1 and A2 samples after friction with different counterparts under C2 conditions. The wear morphology of A1 and A2 did not change significantly compared to those observed without applied voltage. Cracks were present on the worn surfaces of the composites, with lengths of approximately 0.5 mm (Figure 6). Similarly, protrusions were more easily observed on the worn surfaces of sample A1 compared to sample A2, with majority having heights of 100~300 μm. Most of these protrusions on worn surfaces of sample A1 were 100~200 μm in height. As the hardness of the counterpart material increased, the surface roughness of the graphite/copper samples increased accordingly. Similarly, under identical counterpart material conditions, the surface roughness of A1 was higher than that of A2.

**Figure 7:**
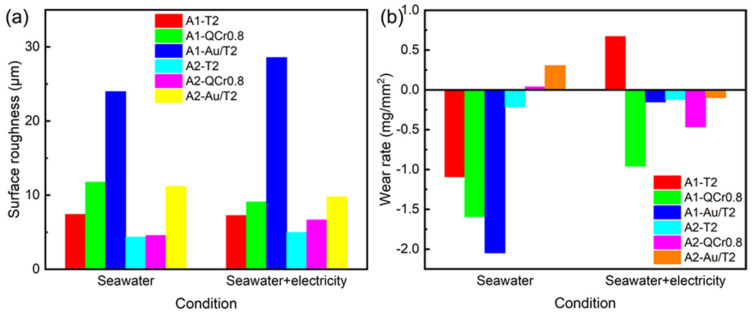
Surface roughness (**a**) and wear rate (**b**) of A1 and A2 samples after friction with T2, QCr0.8, and Au/T2 under C1 and C2 conditions.

To further investigate the morphology changes in the graphite/copper composites under C1 and C2 conditions, the surface roughness of the samples was characterized using a laser confocal microscope. As shown in Figure 7a, the surface roughness of the composites increases with the hardness of the counterpart material, which is consistent with the macroscopic morphology observations. Furthermore, under identical conditions and counterpart materials, the surface roughness of A1 was higher than that of A2. As the hardness of the counterpart material increased, the change in roughness of A2 was less than that of A1. These characterization results demonstrate that as the hardness of the counterpart material increases, the interaction at the contact interface intensifies, leading to greater plastic deformation and the accumulation of wear debris on the sample surfaces, thereby increasing the surface roughness. Reducing the graphite content effectively enhances the resistance of graphite/copper composites to plastic deformation, resulting in more stable roughness changes when interacting with counterparts of varying hardness. Additionally, Figure 2 and Figure 7 illustrate that increased surface roughness may lead to a decrease in the friction coefficient, as the reduced adhesion due to the diminished actual contact area, which is evident in the A1 specimens’ friction behavior [28].

The wear rate is a key indicator of a material tribological behavior and performance. Figure 7b shows the wear rates of A1 and A2 samples after friction with different counterpart materials under conditions C1 and C2. Under the C1 conditions, two graphite/copper samples exhibited negative wear rates, indicating an increase in the post-wear mass of samples A1 and A2. This phenomenon was also observed in C2 conditions. The possible reasons for this include chemical reactions between the copper and substances in the seawater, forming corrosive products, or material transfer from the counterpart disk to the sample surface. Due to the absence of mass loss observed in all A2 specimens under C2 conditions, and considering the importance of controlling wear rates to ensure long-term material durability and stable equipment performance, A2 composites are considered more appropriate for electric contact devices in marine environments.

### 3.4. Microscopic Wear Morphology and Wear Mechanism

To reveal the wear mechanisms of graphite/copper composites under these two conditions, the microscopic wear morphology and surface element distribution of A2 samples were characterized using SEM and EDS.

**Figure 8:**
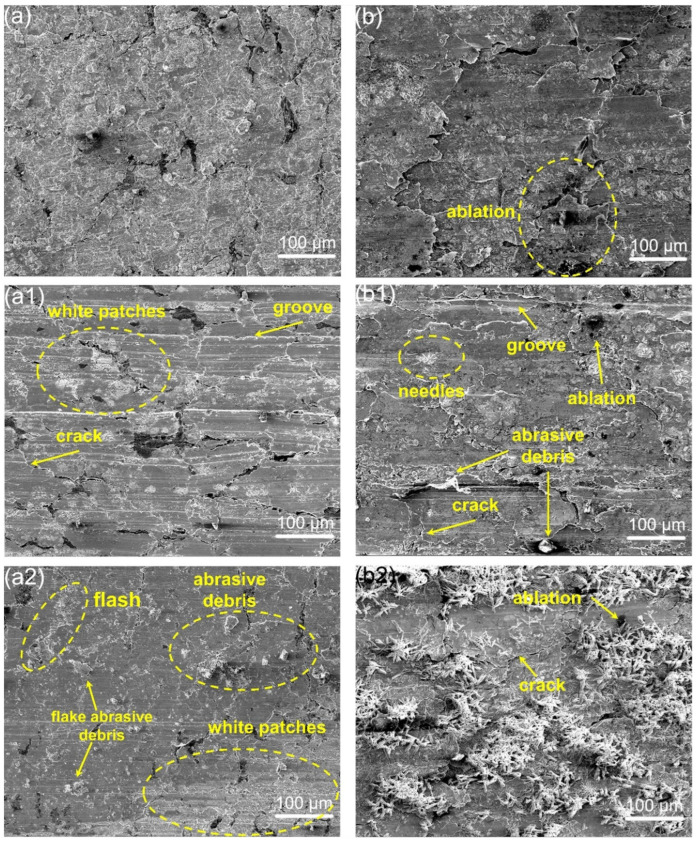
Microscopic wear morphology of A2 samples after friction with T2, QCr0.8, and Au/T2 under C1 (**a**–**a2**) and C2 (**b**–**b2**) conditions.

Figure 8 displays the microscopic wear morphology of the composite under voltage and non-voltage conditions. As depicted in Figure 8a,b, A2 presents various worn morphologies, suggesting different wear mechanisms during friction. Under the C1 conditions, a broken surface was observed, with numerous granular debris evenly distributed across the surface. The wear debris indicates that the A2 composite has experienced abrasive wear. Upon the application of voltage, little debris was found on the worn surface of A2. However, sheet-like stacking was observed on the surface, a characteristic of delamination wear. Concurrently, arc ablation due to electric field breakdown from unstable dynamic contact was observed on the worn surface (Figure 8b).

Figure 8(a1,b1) exhibits morphologies of A2 grounded with QCr0.8 under two operational conditions. Under the non-voltage condition, numerous grooves indicate that abrasive wear remains the primary mechanism of surface damage (Figure 8(a2)). At the same time, some white patches were observed. Under the C2 condition, both sheet-like stacking, crack and abrasive debris indicate simultaneous occurrence of delamination and abrasive wear during the friction of A2. Similarly, damage from arc ablation persisted. Furthermore, it is noteworthy that white patches formed on the A2 worn surface under non-voltage condition, while needle-like structures were observed on the worn surfaces under the voltage condition.

Figure 8(a2,b2) illustrates the worn surfaces of the composite grounded with Au/T2 under both C1 and C2 conditions. For the A2 sample under C1 condition, the presence of flash and flake debris indicates adhesive wear. These granular debris suggest the occurrence of abrasive wear as well. The rolling of debris on the surface induces the appearance of the small pits (Figure 5f). Under C2 conditions, the A2 sample exhibited persistent delamination wear, arc ablation and increasing needle-like substances.

**Figure 9:**
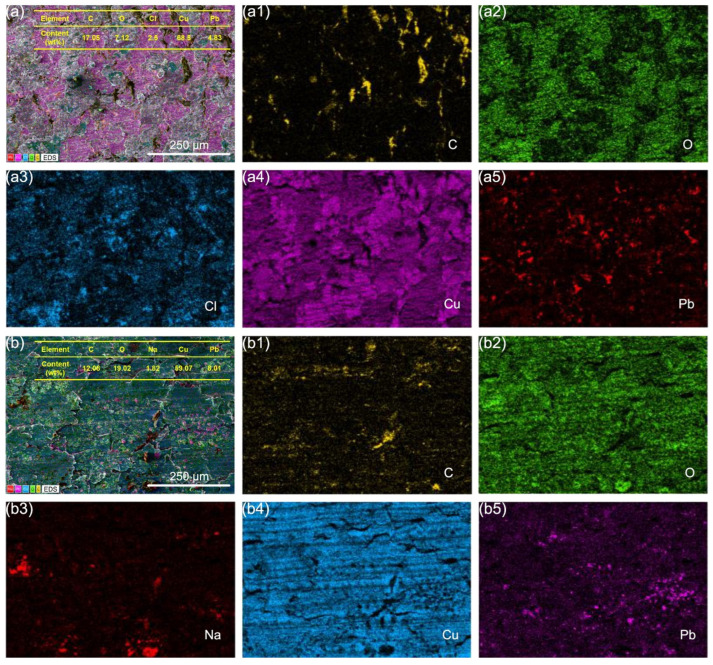
EDS spectra of A2 samples after friction with T2 under C1 (**a**–**a5**) and C2 (**b**–**b5**) conditions.

To further analyze the wear mechanisms of A2 samples, we performed elemental analysis on the wear surfaces of A2 samples after friction with different hardness counterparts under different conditions using EDS. Figure 9 shows the EDS analysis results of the wear surfaces of A2–T2 tribopairs under C1 and C2 conditions. In a seawater environment, the wear surface of A2 mainly contained C, O, Cl, and Pb elements. The graphite phase was separated from Cu matrix under shear forces, resulting in a significant reduction in the relative content of C. As shown in Figure 9(a2–a4), O, Cu, and Cl elements appear to be more concentrated in some regions due to the accumulation of wear debris on the sample surface. The presence of O and Cl elements indicates that the copper matrix reacts chemically with oxygen and chloride ions in the seawater. This confirms that the wear process of A2 involves not only abrasive wear but also corrosion wear. After applying voltage, besides the appearance of Na elements due to the evaporation of residual seawater, the oxygen content on the A2 wear surface significantly increased, while Cl disappeared. This suggests that under C2 conditions, the corrosion type is oxidative corrosion (Figure 9b). Additionally, the ratio of W_(O+Cl)_/W_(Cu+Pb)_ for A2 wear samples under C1 conditions was 0.13, while under C2 conditions, it was W_(O)_/W_(Cu+Pb)_ = 0.28, indicating that the electricity factor accelerates the corrosion of A2 samples. The uniform distribution of C across the entire wear surface of A2 under C2 conditions indicates the formation of a graphite lubricating film during friction.

**Figure 10:**
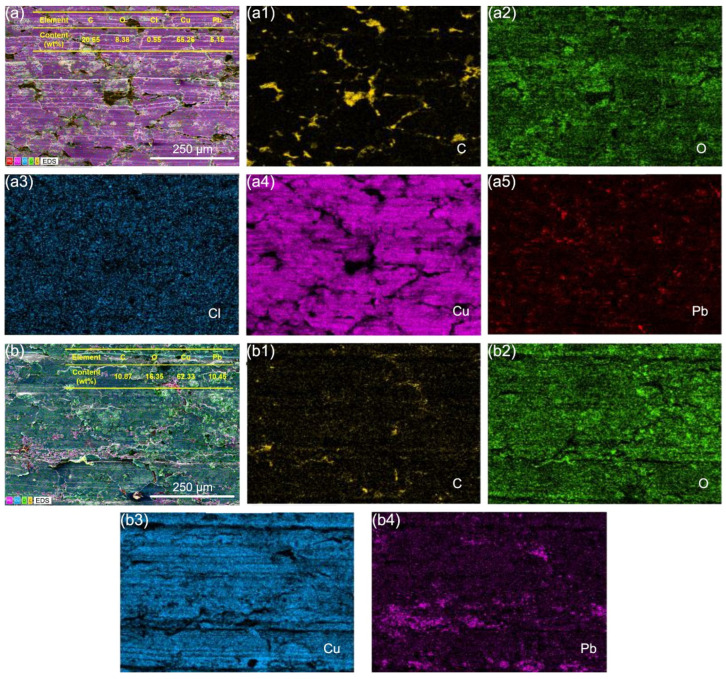
EDS spectra of A2 samples after friction with QCr0.8 under C1 (**a**–**a5**) and C2 (**b**–**b4**) conditions.

Figure 10 compares the elemental distribution on the wear surfaces of A2–QCr0.8 tribopairs under C1 and C2 conditions. After friction with the harder QCr0.8, A2 samples also underwent corrosion reactions, with W_(O+Cl)_/W_(Cu+Pb)_ = 0.13. For the A2–QCr0.8 tribopair under C2 conditions, the oxide content on the A2 wear surface increased, while chlorides nearly disappeared, with W_(O+Cl)_/W_(Cu+Pb)_ = W_(O)_/W_(Cu+Pb)_ = 0.22. The observed phenomenon was consistent with the elemental characterization results of the A2–T2 tribopair under C2 conditions, indicating that although the electric factor accelerates the corrosion of graphite/copper composites, it has opposite effects on the formation of oxygen-containing and chlorine-containing corrosive products. Additionally, as shown in Figure 10(b1), a graphite lubricating layer exists at the contact interface of the A2–QCr0.8 tribopair under C2 conditions.

**Figure 11:**
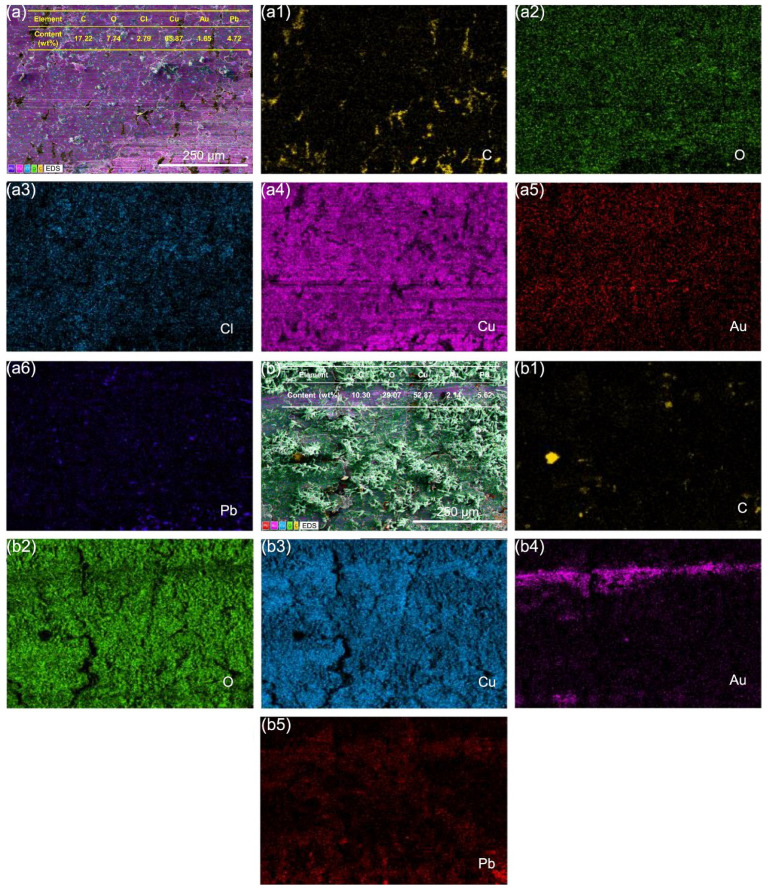
EDS spectra of A2 samples after friction with Au/T2 under C1 (**a**–**a6**) and C2 (**b**–**b5**) conditions.

For the A2–Au/T2 tribopair, the wear surface of A2 samples under C1 conditions showed a similar distribution of O, Cl, Cu, and Pb elements to that observed in the A2–T2 and A2–QCr0.8 tribopairs, with W_(O+Cl)_/W_(Cu+Pb)_ = 0.15. Due to adhesive wear, some Au from the Au/T2 counterpart transferred to the A2 sample surface (Figure 11(a5)). Similarly, as show as Figure 11(b4), Au elements are also observed on the worn surface of A2 under C2 conditions. Furthermore, the weight ratio W_(O+Cl)_/W_(Cu+Pb)_ = W_(O)_/W_(Cu+Pb)_ = 0.50, significantly higher than that of A2 samples in friction with other counterparts under C2 conditions, indicating that the corrosion is further exacerbated. This is because gold has a higher electrode potential than copper in the electrochemical series, making gold more likely to act as the cathode and copper more likely to lose electrons. Additionally, as shown in Figure 11, these unknown substances are oxides/chlorides of copper. Given the minimal distribution of Cl in the region where the unknown substances were located, it is reasonable to infer that these substances were mainly copper oxides. XPS analysis of Cu element reveals that the white patches consist of a mixture of CuO and Cu_2_O, with Cu_2_O as the predominant substance (Figure S1). Similarly, the needle-like objects are also a mixture of CuO and Cu_2_O, predominantly CuO.

## 4. Application Recommendation

This study provides critical insights into optimizing graphite/copper composites for marine electrical systems, addressing urgent challenges in reliability and durability. The findings hold transformative potential for marine engineering applications in extending the service life of critical components, such as subsea connectors, slip ring assemblies and dynamic cable systems, etc. In addition, the study provides an instruction for material pairing optimization. The electrochemical reaction mechanism identified in gold-coated copper pairings provides actionable guidelines for preventing unintended oxide formation in high-reliability marine electronic. This work establishes a framework for designing next-generation marine electrical components, with particular relevance to blue energy infrastructure (wave, tidal and wind power converters), deep-sea exploration equipment and smart port electrification systems. By bridging the knowledge gap in current-carrying tribocorrosion mechanisms, it provides essential data for international standards development in marine material selection.

## 5. Conclusions

In this study, we prepared graphite/copper composites with powder metallurgy to investigate the effects of graphite content, mating materials and electrical voltage on the friction behavior of these composites in a marine environment. The research provides valuable experimental data and theoretical insights into the wear behavior of graphite/copper composites, contributing to their potential application in marine engineering. Based on this research, we draw the following conclusions:

(1) Two graphite/copper composites, differing in graphite content, displayed similar friction coefficients (ranging from 0.2 to 0.3) during the final stage of the friction process in both C1 and C2 conditions.
(2) With the increase in counterpart material hardness, the interaction at the contact interface intensified, resulting in more plastic deformation on the surfaces of the graphite/copper samples, which in turn elevated the surface roughness. Meanwhile, graphite/copper composites of less graphite exhibited lower surface roughness due to higher hardness.
(3) Under C1 and C2 conditions, both graphite/copper samples exhibited a negative wear rate, suggesting that the corrosive products generated during friction tests lead to an increase in the mass of the worn samples instead of decrease.
(4) Under C1 conditions, the composite primarily underwent abrasive wear, while adhesive wear intensifies when grounded with harder Au/T2. Under C2 conditions, delamination wear and arc ablation became predominant, and minor amount of abrasive wear was also present. Similarly, adhesive wear arose when the composite was grounded with Au/T2. Furthermore, corrosive wear, particularly oxidative wear, was observed on the worn surfaces of all specimens.
(5) In a seawater context, the electricity factor not only induced electrical damage to the graphite/copper surface but also aggravated corrosion, leading to an increased oxygen concentration on the wear surface while suppressing the formation of chlorine-containing corrosive substances.

## Figures and Tables

**Table 1:** Chemical element composition of graphite/copper composites.

Material ID	C (wt.%)	Pb (wt.%)	Cu (wt.%)
A1	45	3	52
A2	30	3	67

**Table 2:** Voltage under different operation conditions.

Operation Condition	Voltage (V)
C1	0
C2	3

## Data Availability

The original contributions presented in this study are included in the article/Supplementary Materials. Further inquiries can be directed to the corresponding author.

## Supplementary Materials

The following supporting information can be downloaded at: https://www.mdpi.com/article/10.3390/ma18071516/s1, Figure S1: XPS results of the worn surfaces of A2 after grounded with Au/T2 under C1 (a) and C2 (b) conditions.
