# Supplementary material for: Friction and Wear Performances and Mechanisms of Graphite/Copper Composites Under Electrical Contact in Marine Environments
Source: Materials (Basel). 2025 Mar 28;18(7):1516. doi: 10.3390/ma18071516 (PMC11990517; doi:10.3390/ma18071516)
Supplement: Supplementary file 1 [file materials-18-01516-s001.zip › materials-3463822-supplementary.pdf]

Friction and Wear Performances and Mechanisms of Graphite/Copper Composites Under Electrical Contact in Marine Environments

Nenghui Wang [1,2], Chuanfeng Wang [2], Wenhu Xu [3], Weiping Cheng [2], Haihong Wu [2] and Hongsheng Li [1,*]

1 School of Instrument Science and Engineering, Southeast University, Nanjing 210096, China
2 China Ship Jiujiang Marine Equipment (Group) Co., Ltd., Jiujiang 332008, China
3 School of Advanced Manufacturing, Nanchang University, Nanchang 330031, China
* Correspondence: haiyan_8453@163.com

Experiment Methods

X-ray photoelectron spectroscopy (XPS) was conducted on a Thermo ScientificTM K-AlphaTM+ spectrometer equipped with a monochromatic Al Kα X-ray source (1486.6 eV) operating at 100 W. Samples were analyzed under vacuum ($P < 10{-}8$ mbar) with a pass energy of 150 eV (survey scans) or 50eV (high-resolution scans). All peaks would be calibrated with C1s peak binding energy at 284.8 eV for adventitious carbon.

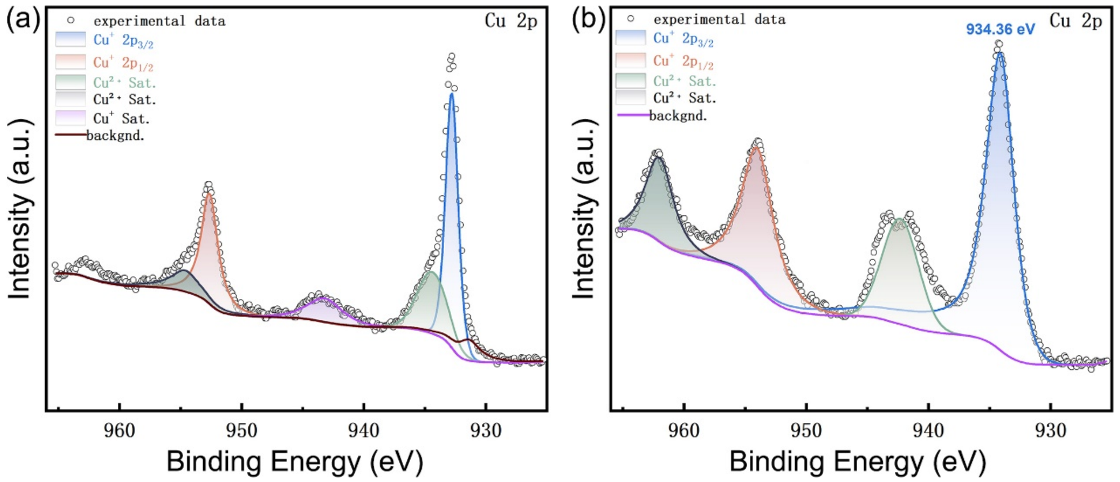

Figure S1. XPS results of the worn surfaces of A2 after grounded with Au/T2 under C1 (a) and C2 (b) conditions.
